# Supplementary material for: Systematic Comparison of Extract Clean-Up with Currently Used Sorbents for Dispersive Solid-Phase Extraction
Source: Molecules. 2024 Sep 30;29(19):4656. doi: 10.3390/molecules29194656 (PMC11478316; doi:10.3390/molecules29194656)
Supplement: Supplementary file 1 [file molecules-29-04656-s001.zip › molecules-3203276-supplementary.pdf]

Systematic Comparison of Extract Clean-Up with Currently Used Sorbents for Dispersive Solid-Phase Extraction

Michelle Peter and Christoph Müller *

Department of Pharmacy, Center for Drug Research, Ludwig-Maximilians-Universität München, 81377 Munich, Germany; michelle.peter@cup.uni-muenchen.de
* Correspondence: christoph.mueller@cup.uni-muenchen.de

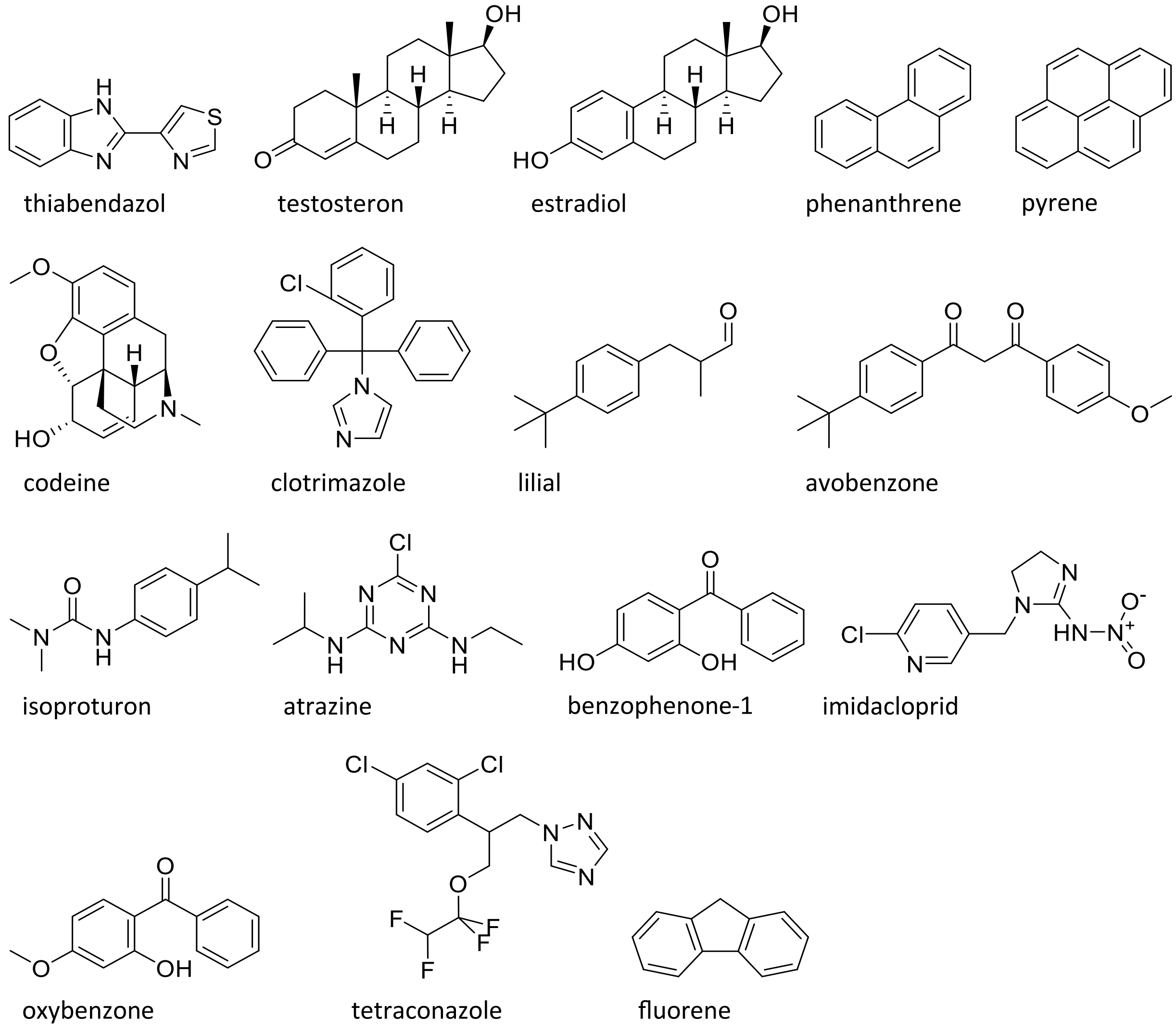

Supplementary Material Figure S1: Structures of analytes mentioned in the text.

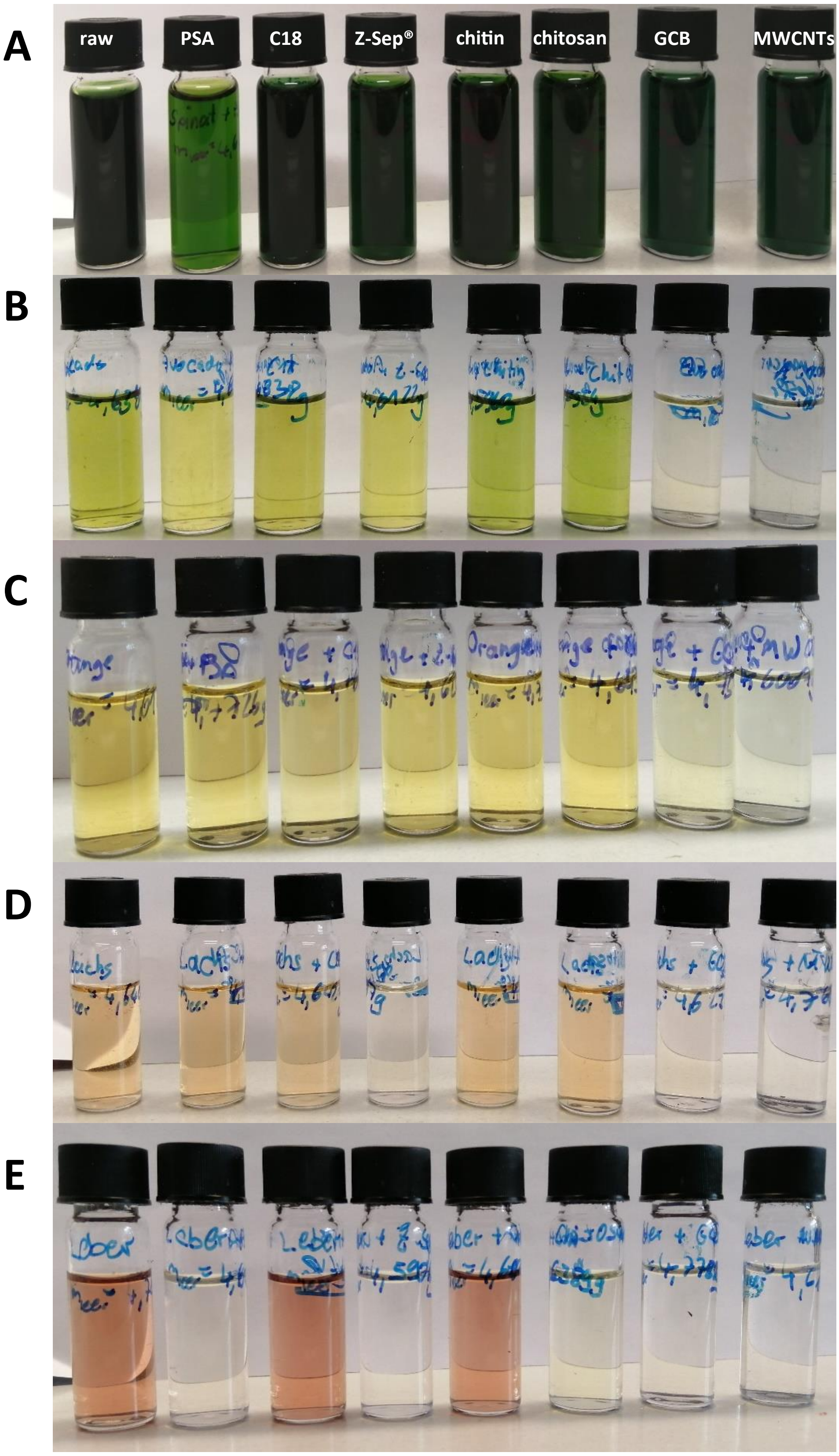

Supplementary Material Figure S2: Colors of extracts of spinach (**A**), avocado (**B**), orange (**C**), salmon (**D**) and liver (**E**) matrix after clean-up with seven different dSPE sorbents

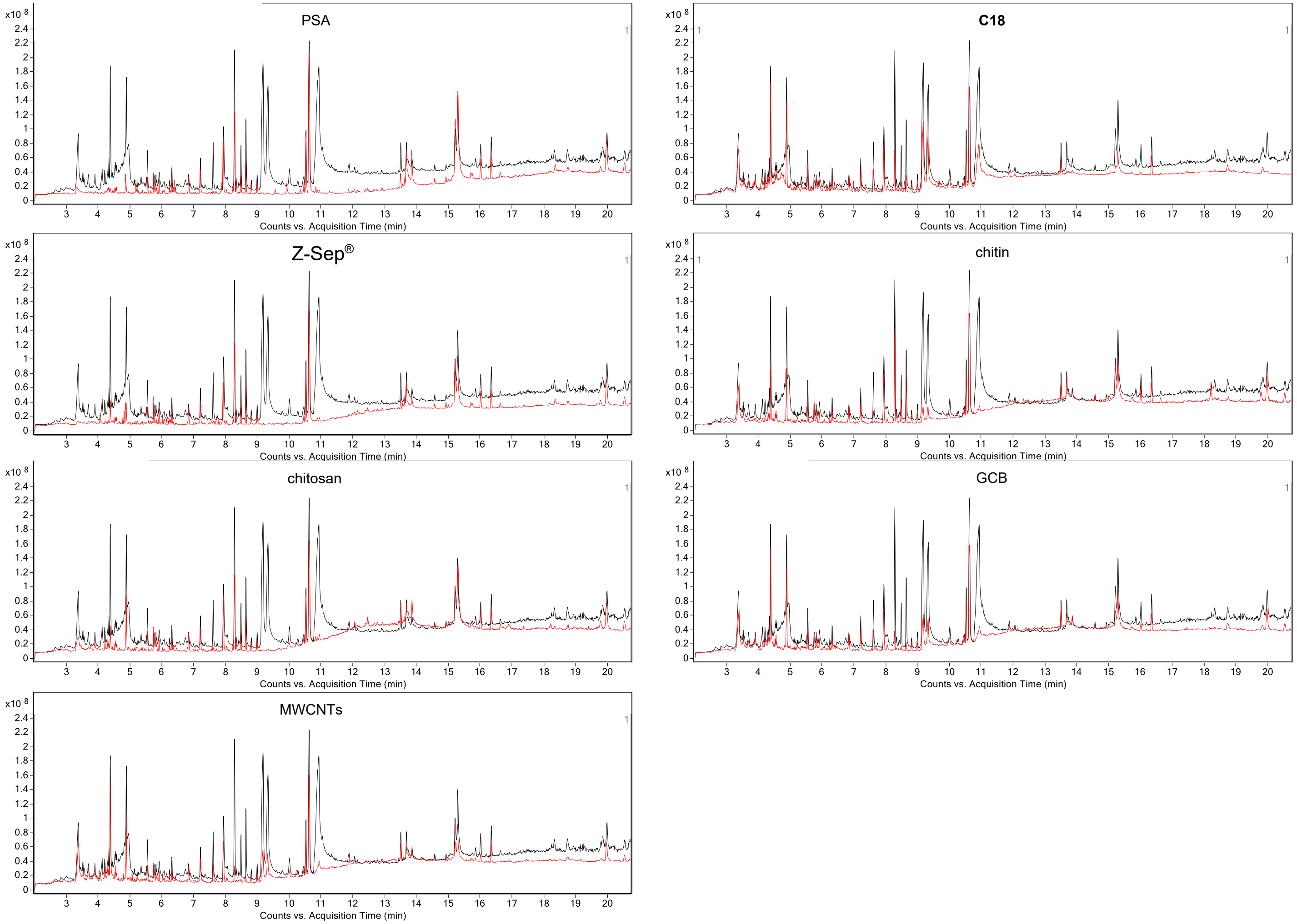
PSA
C18
Z-Sep®
chitin
chitosan
GCB
MWCNTs
x10 8
Counts vs. Acquisition Time (min)

Spinach

Avocado

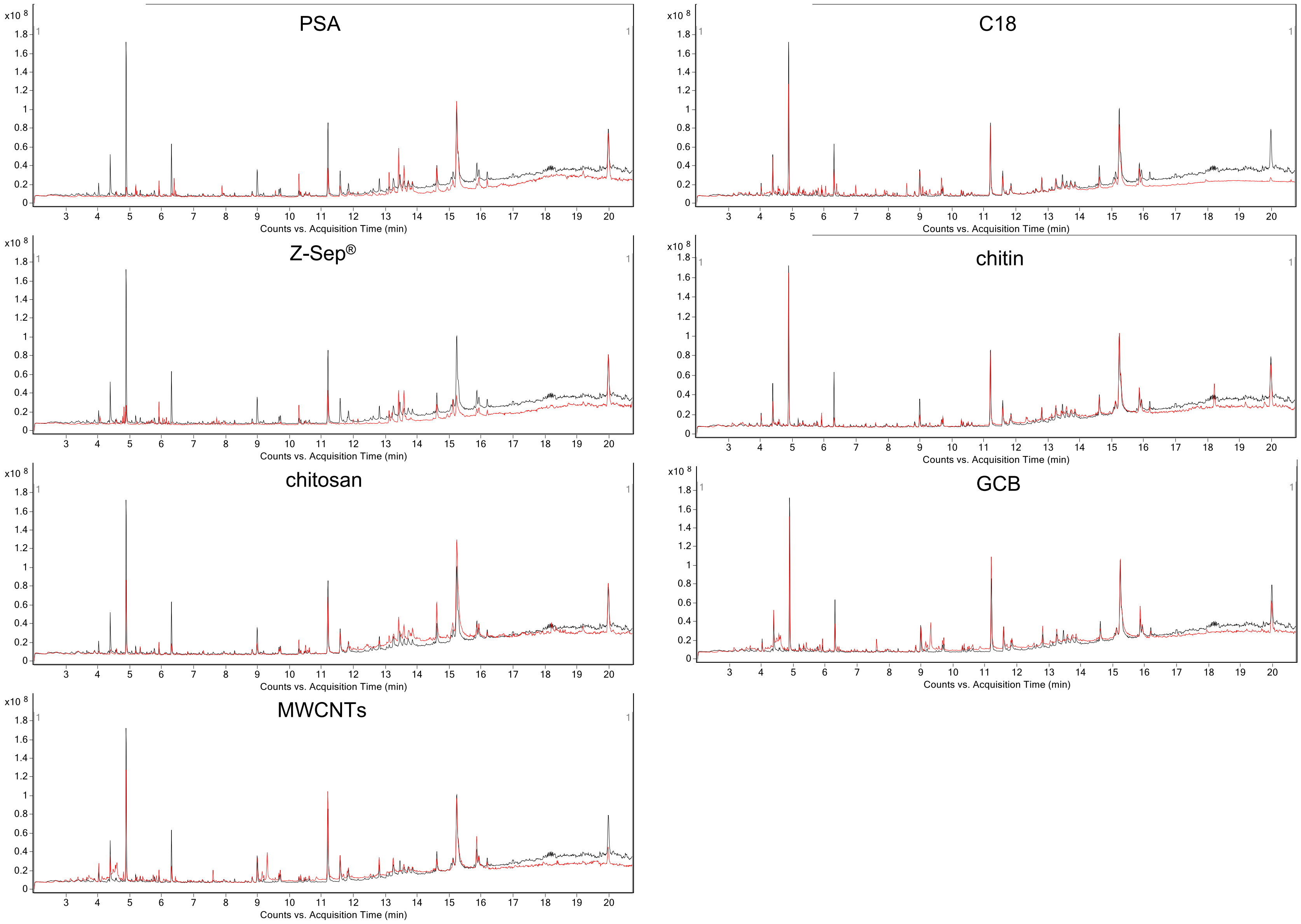

PSA
C18
Z-Sep®
chitin
chitosan
GCB
MWCNTs
x10 8
0
0.2
0.4
0.6
0.8
1
1.2
1.4
1.6
1.8
3 4 5 6 7 8 9 10 11 12 13 14 15 16 17 18 19 20
Counts vs. Acquisition Time (min)

Orange

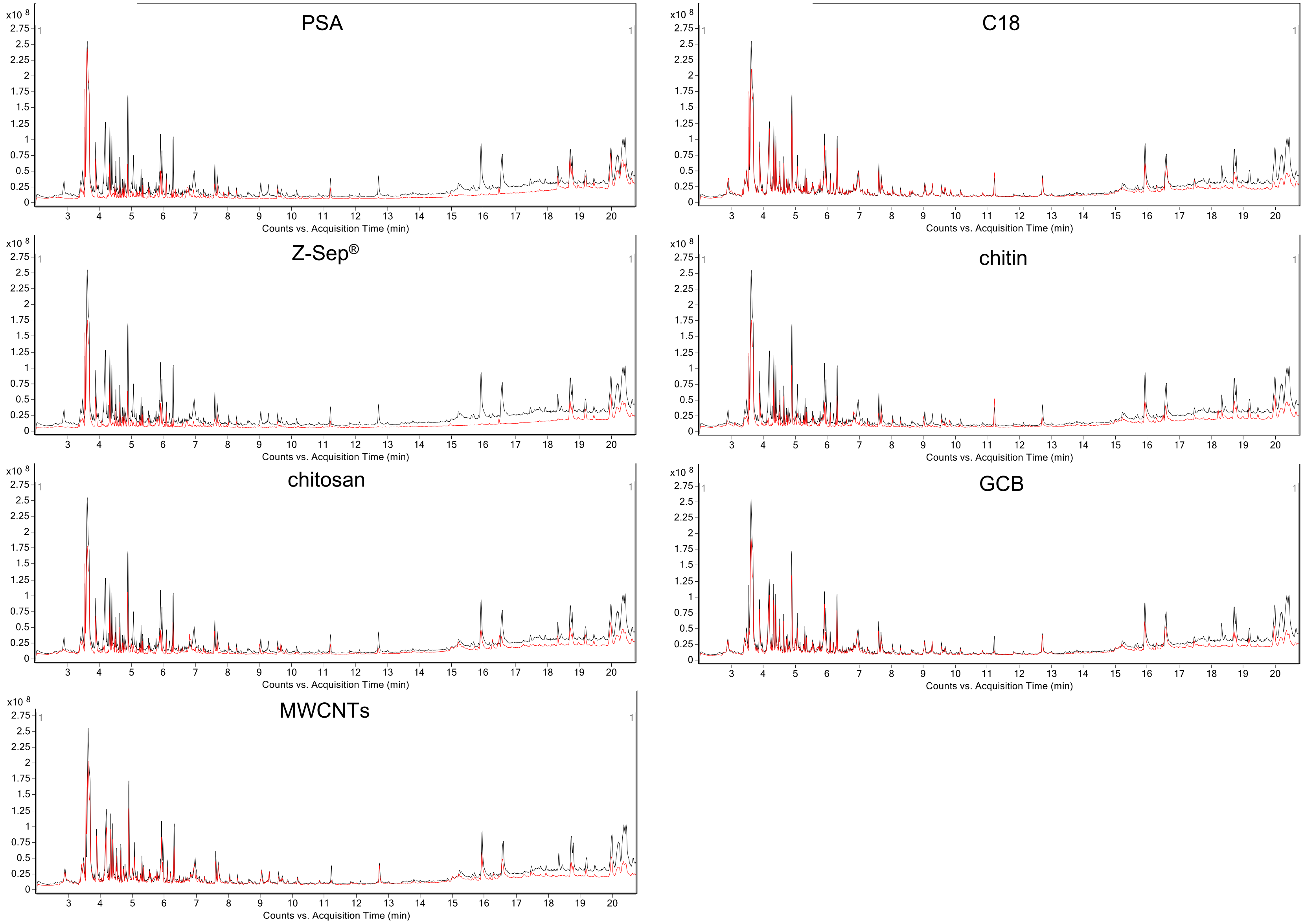
PSA
C18
Z-Sep®
chitin
chitosan
GCB
MWCNTs
x10 8
Counts vs. Acquisition Time (min)

Salmon

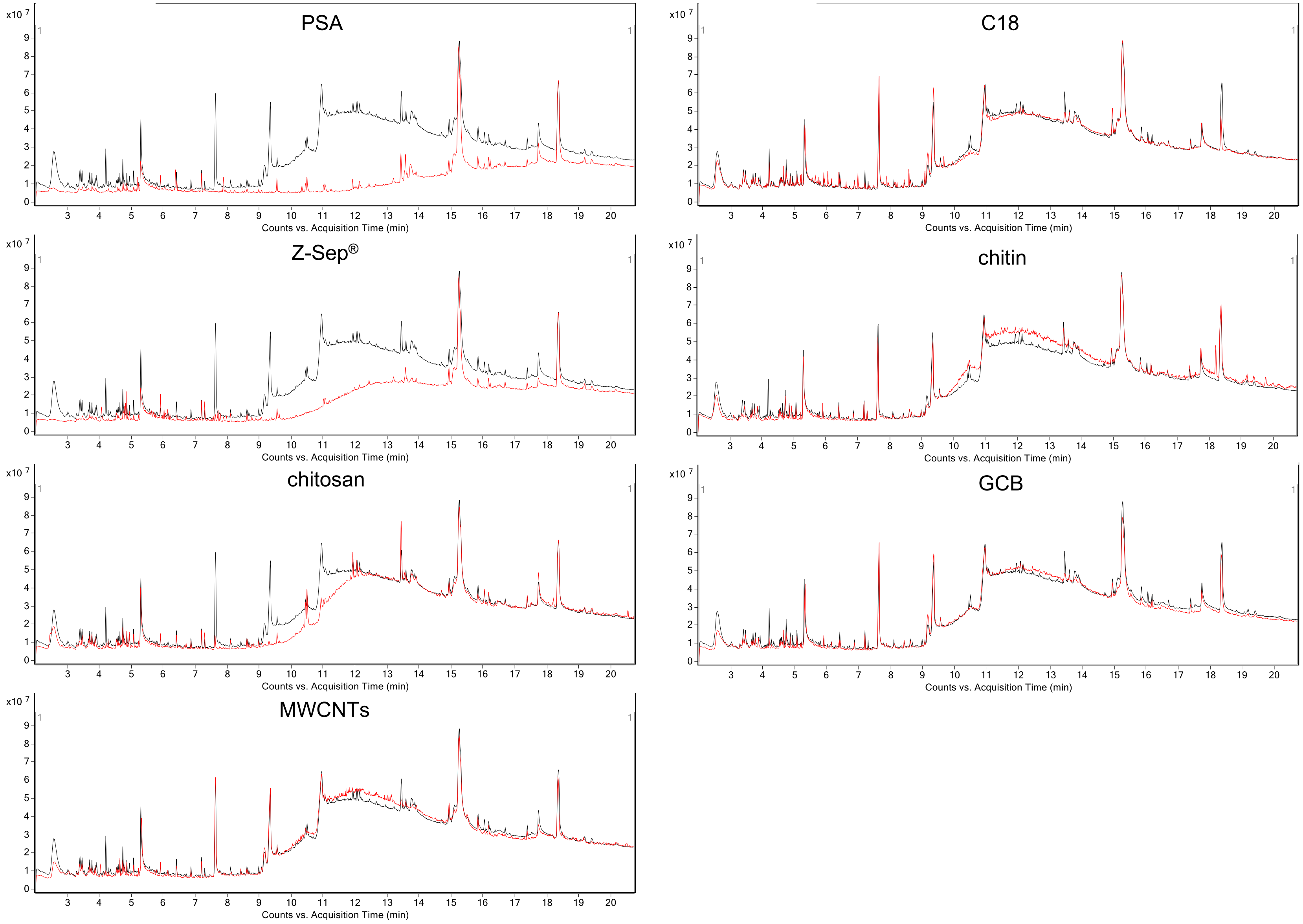

PSA
C18
Z-Sep®
chitin
chitosan
GCB
MWCNTs
x10 7
Counts vs. Acquisition Time (min)

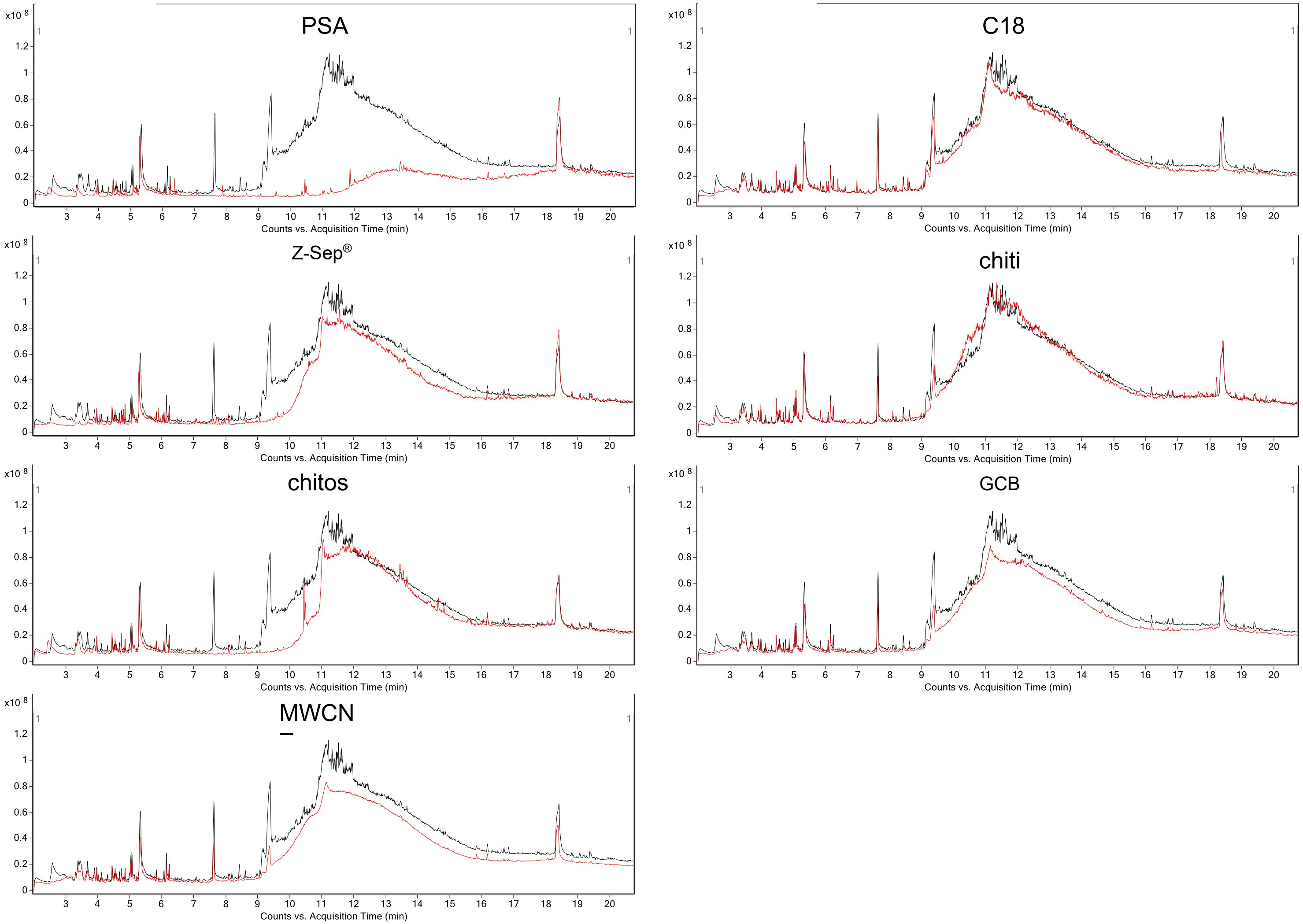

Liver

Supplementary Material Figure S3: TIC chromatograms of GC-MS scan data (*m/z* 100-600) of raw (**black**) and cleaned-up (**red**) matrix extracts.

Supplementary Material Table S1: Peak areas of TIC (*m/z* 100-600) of matrix extracts cleaned-up with seven different sorbents (PSA, C18, Z-Sep®, chitin, chitosan, GCB and MWCNTs. The peak area was determined by using Agilent's Agile2 integration algorithm in the Qualitative Analysis Navigator program (version B.08.00 from 2016).; n = 3, mean given in counts (absolute) or % (relative)).

absolute	raw	PSA	C18	Z-Sep®	chitin	chitosan	GCB	MWCNTs
spinach	8294761680	3678531474	4754955654	2787809522	2886117965	3672808385	3508451246	3306802207
avocado	1988129494	1431585147	1633883975	1128169480	1968604209	1928172165	2246046162	2092327412
orange	7273021923	4216837101	5926059853	3377104572	4060193109	4429921920	5288906624	5159550821
salmon	2164454280	1385899947	1953150408	1231264031	1860369419	1720674945	1744894085	1588940569
liver	2919845834	1121296776	1814608159	920749063	1760423787	1560738923	1384766228	992413918

relative	raw	PSA	C18	Z-Sep®	chitin	chitosan	GCB	MWCNTs
spinach	100	44	57	34	35	44	42	40
avocado	100	72	82	57	99	97	113	105
orange	100	58	81	46	56	61	73	71
salmon	100	64	90	57	86	79	81	73
liver	100	38	62	32	60	53	47	34

Supplementary Material Table S2: List of all analytes with their recoveries in the respective matrices (n = 3, mean given in %). Recoveries below 70% are indicated in ***red***.

Matrix: Spinach	PSA	C18	Z-Sep®	chitin	chitosan	GCB	MWCNTs
acenaphthene	102	110	117	103	114	128	73
atrazine	79	87	84	83	85	96	87
avobenzone	75	85	***48***	88	90	95	***61***
azoxystrobin	89	90	92	87	90	104	93
BDE-99	82	86	94	88	89	98	89
benzophenone-1	73	102	90	80	93	99	73
BHC-β	101	113	120	110	110	113	99
BHT	99	115	122	108	99	120	111
bifenthrin	77	97	95	89	89	99	91
boscalid	93	103	105	102	101	103	102
caffeine	83	100	86	84	88	93	83
carbetamide	85	110	100	83	84	103	90
carbofuran	85	116	101	87	89	106	93
celestolide	89	103	102	94	96	111	96
chlordane-trans	85	111	99	94	92	101	91
chlorpyrifos	79	106	91	88	82	102	90
chlorpyrifos-methyl	79	96	99	89	91	108	92
climbazole	80	102	91	85	89	98	87
clopidogrel	84	104	95	87	90	101	91
clotrimazole	***66***	95	77	72	84	98	90
codeine	79	102	***65***	***68***	86	98	90
cybutryne	80	98	94	87	90	99	91
cyflufenamid	83	101	94	87	85	97	88
cyhalothrin-λ	93	90	91	83	88	97	92
DDE-*p,p'*	77	102	98	91	91	101	91
DEHA	87	91	95	90	88	99	91
DEHTP	90	79	94	94	91	100	93

Matrix: Spinach	PSA	C18	Z-Sep®	chitin	chitosan	GCB	MWCNTs
deltamethrin	102	84	95	85	93	104	96
desmedipham	87	105	99	77	91	93	81
diazepam	80	100	91	85	89	99	92
dicofol-*o, p'*	82	106	98	92	87	101	75
dieldrin	79	104	97	94	91	104	93
difenoconazole	96	86	87	86	87	100	95
diflubenzuron	80	92	102	81	87	90	***62***
dimethomorph	91	96	93	83	92	100	95
diphenylamine	84	109	111	102	100	110	93
EHS	86	105	104	97	99	115	96
epoxiconazole	94	106	100	89	88	98	88
17 β-estradiol	85	95	93	81	91	100	90
17α-ethinylestradiol	84	93	92	77	87	98	92
fenhexamid	73	106	89	81	91	100	89
fenpropidin	77	93	78	80	86	96	87
fenpropimorph	85	103	83	79	80	101	92
fenvalerate	95	89	94	84	91	102	93
fipronil	84	99	95	88	91	99	94
fipronil sulfone	79	103	95	80	87	97	89
fluazifop-p-butyl	81	102	96	87	87	99	90
fludioxonil	78	106	97	90	88	97	86
fluopyram	84	99	94	86	89	98	90
fluorene	93	109	124	110	108	116	***58***
flupyradifurone	83	91	86	84	80	101	90
fluvalinat-*τ*	96	86	93	82	91	99	95
galaxolide	82	103	100	93	94	109	92
heptachlor	114	114	141	114	111	116	105

Matrix: Spinach	PSA	C18	Z-Sep®	chitin	chitosan	GCB	MWCNTs
imidacloprid	81	93	98	82	79	94	78
isoproturon	76	***59***	91	88	92	111	103
lenacil	80	107	93	86	92	99	91
lilial	***52***	88	98	92	91	98	95
mestranol	79	96	101	84	91	100	91
metazachlor	85	97	94	89	90	102	90
metrafenone	88	94	93	87	91	96	90
miconazol	91	87	82	79	87	97	75
myclobutanil	79	105	95	86	88	98	90
octocrylene	85	93	90	87	90	98	93
4-*tert*-octylphenol	82	98	101	92	93	103	92
oxybenzone	82	103	91	90	91	100	81
PCB 101	88	89	96	97	92	96	89
PCB 138	79	91	95	92	89	98	89
PCB 153	78	95	98	90	88	98	87
PCB 180	82	85	96	92	89	97	88
PCB 28	80	101	107	95	96	107	87
PCB 52	84	98	98	95	101	105	94
pentachloronitrobenzene	90	105	118	102	104	116	101
permethrin	82	90	95	84	91	96	93
phenanthrene	87	106	113	101	103	105	***30***
picolinafen	80	99	94	87	90	96	70
pirimicarb	78	98	90	84	88	98	91
propiconazole	80	106	90	86	88	99	88
pyrene	86	90	92	92	92	77	***10***
pyrimethanil	82	100	97	90	92	93	***70***
quinoxyfen	81	99	87	89	88	95	72
simazine	71	94	87	82	88	96	83
spirodiclofen	79	92	93	85	85	102	89
TDCPP	84	109	91	87	90	97	91

Matrix: Spinach	PSA	C18	Z-Sep®	chitin	chitosan	GCB	MWCNTs
tebuconazole	83	104	91	86	88	99	88
terbuthylazine	78	89	88	84	89	98	89
terbuthylazine-desethyl	72	80	77	80	81	94	85
terbutryn	83	102	93	86	92	90	85
testosterone	73	96	93	85	90	101	88
tetraconazole	80	107	91	85	82	97	89
thiabendazole	81	96	97	82	88	***69***	***24***
tolclofos-methyl	79	102	101	94	91	107	92
tonalid sum	83	102	99	92	93	108	93
triclosan	81	109	95	87	90	99	89
triphenyl phosphate	85	104	93	87	89	99	90
uvinul a plus	94	87	91	86	90	101	92
verapamil	99	92	77	79	88	105	98
zoxamide	90	95	93	90	84	108	88

Matrix: Avocado	PSA	C18	Z-Sep®	chitin	chitosan	GCB	MWCNTs
acenaphthene	117	117	111	123	112	108	***45***
atrazine	104	109	103	99	103	107	92
avobenzone	90	98	***17***	101	104	***66***	***9***
azoxystrobin	106	113	114	100	104	122	106
BDE-99	99	98	105	102	100	102	84
benzophenone-1	86	104	***69***	96	100	100	***57***
BHC-β	106	110	118	124	118	118	92
BHT	105	124	126	115	111	129	100
bifenthrin	100	105	106	100	101	113	101
boscalid	104	112	109	99	102	114	83
caffeine	97	102	99	102	98	97	72
carbetamide	110	106	106	96	107	109	92
carbofuran	104	106	102	99	104	106	90
celestolide	105	108	117	113	107	116	97
chlordane-trans	104	103	109	106	107	115	98
chlorpyrifos	102	105	110	100	102	106	91
chlorpyrifos-methyl	105	108	111	106	109	114	95
climbazole	96	106	104	96	97	109	85
clopidogrel	101	106	108	102	103	115	99
clotrimazole	79	99	91	104	87	75	74
codeine	98	109	74	85	100	113	94
cybutryne	99	106	107	101	101	109	92
cyflufenamid	101	109	107	99	96	155	99
cyhalothrin-λ	102	113	106	101	96	116	105
DDE-*p,p'*	100	102	107	102	97	104	90
DEHA	100	93	103	100	101	113	101
DEHTP	99	83	107	99	98	105	99
deltamethrin	102	107	108	98	104	119	108
desmedipham	95	105	100	103	108	110	77
diazepam	99	106	104	100	100	112	97

Matrix: Avocado	PSA	C18	Z-Sep®	chitin	chitosan	GCB	MWCNTs
dicofol-*o, p'*	98	106	108	107	105	107	***60***
dieldrin	103	107	107	98	101	109	92
difenoconazole	106	112	110	97	103	119	99
diflubenzuron	91	103	98	93	94	98	***26***
dimethomorph	105	116	108	97	106	118	107
diphenylamine	105	121	122	118	113	125	84
EHS	108	106	113	114	106	114	93
epoxiconazole	103	106	99	100	102	111	84
17 β-estradiol	101	110	105	98	101	110	83
17α-ethinylestradiol	100	110	108	96	100	112	98
fenhexamid	86	108	93	98	102	112	92
fenpropidin	103	107	104	90	97	107	97
fenpropimorph	101	108	104	94	99	108	96
fenvalerate	102	110	109	98	104	118	108
fipronil	101	107	105	104	103	114	96
fipronil sulfone	99	107	104	97	97	111	96
fluazifop-p-butyl	100	105	105	100	100	109	95
fludioxonil	93	105	100	100	95	101	75
fluopyram	100	106	107	100	101	111	94
fluorene	112	122	127	114	113	113	***28***
flupyradifurone	99	117	105	99	99	109	93
fluvalinat-τ	107	112	109	99	102	126	106
galaxolide	105	105	114	109	105	116	98
heptachlor	119	112	126	142	122	129	98
imidacloprid	106	112	***63***	83	80	120	84
isoproturon	110	97	108	94	104	113	102
lenacil	98	106	105	100	100	111	92
lilial	74	121	116	114	105	119	96
mestranol	99	105	106	95	100	107	95
metazachlor	101	105	104	98	104	110	94

Matrix: Avocado	PSA	C18	Z-Sep®	chitin	chitosan	GCB	MWCNTs
metrafenone	104	107	110	99	102	110	94
miconazol	101	114	105	94	102	111	***31***
myclobutanil	100	106	104	98	98	108	91
octocrylene	100	105	113	103	99	115	99
4-*tert*-octylphenol	100	104	106	102	99	111	91
oxybenzone	97	105	74	106	102	108	***66***
PCB 101	101	100	106	105	106	112	92
PCB 138	100	100	103	102	98	101	90
PCB 153	102	97	105	104	97	106	92
PCB 180	100	96	105	102	101	109	93
PCB 28	105	107	116	114	112	114	79
PCB 52	105	102	109	109	103	112	87
pentachloronitrobenzene	117	112	141	117	125	120	88
permethrin	101	106	107	102	100	110	97
phenanthrene	107	111	123	117	114	79	***5***
picolinafen	100	107	108	101	100	99	***18***
pirimicarb	93	103	103	100	100	103	89
propiconazole	100	105	106	98	100	109	93
pyrene	100	102	107	106	99	***28***	***0***
pyrimethanil	97	104	106	104	100	91	***33***
quinoxyfen	100	102	106	101	99	96	***37***
simazine	89	104	99	98	102	101	85
spirodiclofen	100	105	107	96	103	118	110
TDCPP	101	106	105	98	100	114	101
tebuconazole	101	106	104	99	101	113	86
terbuthylazine	102	108	105	101	102	107	94
terbuthylazine-desethyl	103	107	97	98	101	101	88
terbutryn	99	100	102	98	96	101	83
testosterone	106	106	108	95	102	110	85
tetraconazole	99	104	105	98	99	108	90

Matrix: Avocado	PSA	C18	Z-Sep®	chitin	chitosan	GCB	MWCNTs
thiabendazole	103	106	100	99	96	***68***	***3***
tolclofos-methyl	104	107	111	106	106	112	92
tonalid sum	104	104	113	109	104	114	96
triclosan	95	107	105	102	100	109	90
triphenyl phosphate	101	107	108	101	100	113	95
uvinul a plus	103	112	88	101	104	119	114
verapamil	110	124	116	93	104	134	105
zoxamide	108	115	101	102	110	111	105

Matrix: Orange	PSA	C18	Z-Sep®	chitin	chitosan	GCB	MWCNTs
acenaphthene	110	126	125	109	114	125	***61***
atrazine	87	96	95	96	98	94	95
avobenzone	89	93	73	98	102	97	***51***
azoxystrobin	95	99	100	96	101	103	95
BDE-99	92	90	96	96	99	100	93
benzophenone-1	74	101	93	93	89	99	88
BHC-β	92	102	106	88	95	113	105
BHT	122	124	120	108	118	129	111
bifenthrin	96	95	94	94	99	102	100
boscalid	92	98	94	95	100	100	89
caffeine	92	97	93	99	95	102	86
carbetamide	97	104	94	98	96	103	102
carbofuran	93	98	101	94	100	102	100
celestolide	100	103	108	101	109	117	106
chlordane-trans	93	93	99	93	97	108	97
chlorpyrifos	90	101	99	95	105	103	96
chlorpyrifos-methyl	94	101	102	97	107	103	95
climbazole	95	95	89	96	100	105	97
clopidogrel	96	98	97	96	102	106	105
clotrimazole	88	97	90	96	106	111	99
codeine	91	102	***66***	84	101	103	97
cybutryne	95	105	96	96	100	105	97
cyflufenamid	91	103	94	96	100	95	96
cyhalothrin-λ	101	97	91	97	102	106	101
DDE-*p,p'*	95	91	98	96	101	109	99
DEHA	100	86	93	96	101	103	105
DEHTP	99	79	91	98	113	107	97
deltamethrin	95	95	92	92	93	99	93
desmedipham	99	105	101	95	92	98	92
diazepam	91	101	93	94	98	103	98

Matrix: Orange	PSA	C18	Z-Sep®	chitin	chitosan	GCB	MWCNTs
dicofol-*o, p'*	93	102	100	99	104	106	75
dieldrin	94	93	96	95	100	112	100
difenoconazole	94	98	90	93	100	103	92
diflubenzuron	104	98	96	97	97	96	***49***
dimethomorph	93	95	94	97	98	104	94
diphenylamine	102	120	115	106	123	150	107
EHS	102	99	107	102	108	115	103
epoxiconazole	91	97	90	92	92	100	99
17 β-estradiol	95	100	94	97	98	102	90
17α-ethinylestradiol	94	101	92	96	101	105	95
fenhexamid	81	98	94	92	101	105	102
fenpropidin	91	100	87	95	101	103	96
fenpropimorph	94	103	91	96	105	104	99
fenvalerate	94	97	94	93	94	102	95
fipronil	97	106	95	91	97	102	98
fipronil sulfone	96	96	94	95	99	106	97
fluazifop-p-butyl	97	98	96	96	98	107	98
fludioxonil	95	98	94	96	99	106	92
fluopyram	97	103	94	95	98	104	96
fluorene	114	114	120	108	115	116	***43***
flupyradifurone	90	104	99	102	99	105	101
fluvalinat-*τ*	94	97	95	97	97	100	96
galaxolide	99	102	106	101	110	115	104
heptachlor	94	104	105	88	90	115	108
imidacloprid	83	84	83	87	118	118	103
isoproturon	84	86	92	89	91	90	84
lenacil	94	100	95	95	100	109	101
lilial	84	109	106	102	110	118	101
mestranol	91	99	94	97	98	102	97
metazachlor	93	103	95	91	96	99	94

Matrix: Orange	PSA	C18	Z-Sep®	chitin	chitosan	GCB	MWCNTs
metrafenone	93	94	95	94	97	103	97
miconazol	91	101	81	95	102	100	77
myclobutanil	95	96	94	96	99	107	97
octocrylene	92	96	93	95	101	101	97
4-*tert*-octylphenol	98	99	100	97	101	107	101
oxybenzone	95	104	94	97	101	106	85
PCB 101	92	94	103	96	111	111	94
PCB 138	93	87	95	93	101	103	105
PCB 153	95	91	96	96	101	106	99
PCB 180	92	89	96	95	101	97	97
PCB 28	97	104	109	107	113	118	95
PCB 52	95	103	105	102	112	118	107
pentachloronitrobenzene	108	107	121	97	101	118	105
permethrin	101	100	90	94	97	100	92
phenanthrene	104	107	113	106	110	94	***12***
picolinafen	96	99	93	95	98	98	***67***
pirimicarb	90	100	96	96	104	102	95
propiconazole	95	98	93	93	100	105	106
pyrene	97	92	97	96	103	***58***	***4***
pyrimethanil	96	100	98	96	102	98	***69***
quinoxyfen	92	93	93	94	99	98	74
simazine	88	90	103	96	103	88	94
spirodiclofen	90	98	91	89	98	96	94
TDCPP	96	99	94	95	97	103	100
tebuconazole	95	98	93	95	98	103	103
terbuthylazine	89	96	96	93	99	94	90
terbuthylazine-desethyl	82	95	96	95	98	93	89
terbutryn	89	102	96	95	105	104	97
testosterone	89	98	95	97	103	96	92
tetraconazole	92	102	93	96	101	103	96

Matrix: Orange	PSA	C18	Z-Sep®	chitin	chitosan	GCB	MWCNTs
thiabendazole	97	106	87	91	96	***69***	***13***
tolclofos-methyl	93	101	100	99	106	101	97
tonalid sum	97	103	105	100	109	114	103
triclosan	94	97	96	94	100	103	99
triphenyl phosphate	96	99	95	96	98	101	101
uvinul a plus	99	97	95	96	102	106	96
verapamil	99	110	90	99	102	108	100
zoxamide	81	91	89	87	79	85	96

Matrix: Salmon	PSA	C18	Z-Sep®	chitin	chitosan	GCB	MWCNTs
acenaphthene	111	112	130	131	122	113	***28***
atrazine	97	***59***	102	102	102	93	95
avobenzone	***67***	83	***12***	106	99	***64***	***10***
azoxystrobin	82	91	97	104	103	94	89
BDE-99	87	86	103	106	105	93	84
benzophenone-1	72	87	***68***	86	77	81	***49***
BHC-β	94	94	113	111	114	111	97
BHT	109	109	132	121	113	112	113
bifenthrin	85	91	101	106	105	96	93
boscalid	84	92	95	103	102	93	83
caffeine	86	90	99	104	104	95	84
carbetamide	85	91	106	101	106	98	88
carbofuran	87	95	111	99	103	94	90
celestolide	96	98	117	114	112	108	101
chlordane-trans	87	92	110	108	107	101	94
chlorpyrifos	89	90	113	109	107	99	94
chlorpyrifos-methyl	90	94	106	105	110	104	95
climbazole	85	95	103	103	106	95	89
clopidogrel	85	94	103	107	106	97	93
clotrimazole	88	100	101	108	110	88	89
codeine	82	96	***68***	95	108	96	93
cybutryne	85	86	98	107	104	97	93
cyflufenamid	84	96	105	104	108	100	96
cyhalothrin-λ	84	98	95	110	103	95	93
DDE-*p,p'*	88	94	109	109	107	99	94
DEHA	83	82	102	106	104	96	94
DEHTP	87	74	101	105	104	92	89
deltamethrin	85	86	103	100	103	93	90
desmedipham	84	91	96	95	100	95	74
diazepam	86	94	106	104	103	92	89

Matrix: Salmon	PSA	C18	Z-Sep®	chitin	chitosan	GCB	MWCNTs
dicofol-*o, p'*	88	92	111	110	107	100	***59***
dieldrin	88	96	106	110	103	99	96
difenoconazole	80	86	87	98	100	93	88
diflubenzuron	88	94	100	105	102	86	***8***
dimethomorph	81	89	86	102	103	89	87
diphenylamine	97	105	124	117	115	111	80
EHS	95	93	110	115	112	111	98
epoxiconazole	83	93	95	104	103	95	90
17 β-estradiol	82	92	93	104	103	91	83
17α-ethinylestradiol	85	93	97	105	103	94	87
fenhexamid	73	93	84	109	107	97	91
fenpropidin	83	96	93	104	104	90	90
fenpropimorph	86	94	104	107	103	98	95
fenvalerate	82	88	93	100	102	94	92
fipronil	83	87	96	105	105	96	93
fipronil sulfone	84	95	102	109	104	97	94
fluazifop-p-butyl	84	94	105	107	104	95	93
fludioxonil	85	97	104	106	106	96	78
fluopyram	84	86	95	104	104	95	92
fluorene	104	109	135	119	119	102	***13***
flupyradifurone	81	88	91	98	101	86	86
fluvalinat-τ	83	89	91	103	101	95	91
galaxolide	95	93	112	113	110	107	99
heptachlor	90	98	121	113	110	102	94
imidacloprid	88	102	94	101	98	86	95
isoproturon	89	84	107	93	101	91	93
lenacil	84	96	105	107	108	99	95
lilial	***62***	106	127	122	116	116	105
mestranol	87	94	97	103	104	93	90
metazachlor	84	89	97	103	104	95	93

Matrix: Salmon	PSA	C18	Z-Sep®	chitin	chitosan	GCB	MWCNTs
metrafenone	86	94	97	109	108	91	94
miconazol	82	88	92	101	105	81	***34***
myclobutanil	84	96	101	107	106	96	94
octocrylene	85	90	101	104	106	95	92
4-*tert*-octylphenol	90	91	110	107	106	99	95
oxybenzone	85	90	***68***	107	105	99	***68***
PCB 101	91	91	101	105	106	96	89
PCB 138	83	88	109	106	106	97	92
PCB 153	85	89	116	100	103	89	86
PCB 180	84	83	107	107	105	93	88
PCB 28	97	97	115	117	113	109	77
PCB 52	94	100	111	107	111	108	97
pentachloronitrobenzene	99	99	122	109	112	112	92
permethrin	89	93	103	106	105	94	96
phenanthrene	97	101	116	116	115	***67***	***2***
picolinafen	85	96	106	102	106	89	***14***
pirimicarb	86	89	101	104	104	96	92
propiconazole	84	94	104	108	105	96	92
pyrene	88	96	109	106	108	***19***	***0***
pyrimethanil	86	90	102	105	105	86	***32***
quinoxyfen	85	90	106	106	106	90	***35***
simazine	87	96	108	99	107	92	88
spirodiclofen	83	90	110	102	101	96	96
TDCPP	83	97	108	104	105	95	94
tebuconazole	84	94	99	109	106	97	89
terbuthylazine	85	93	99	102	104	94	92
terbuthylazine-desethyl	80	98	100	99	103	91	92
terbutryn	85	91	100	102	104	94	85
testosterone	83	88	99	100	107	90	86
tetraconazole	85	92	103	106	103	94	91

Matrix: Salmon	PSA	C18	Z-Sep®	chitin	chitosan	GCB	MWCNTs
thiabendazole	83	83	93	98	102	70	***5***
tolclofos-methyl	89	94	107	105	109	100	93
tonalid sum	94	93	109	111	109	105	98
triclosan	84	86	98	107	105	97	93
triphenyl phosphate	84	94	102	106	105	97	93
uvinul a plus	85	90	74	104	108	95	91
verapamil	79	88	77	100	99	86	82
zoxamide	78	88	96	93	105	94	96

Matrix: Liver	PSA	C18	Z-Sep®	chitin	chitosan	GCB	MWCNTs
acenaphthene	113	115	109	116	103	126	***47***
atrazine	97	101	101	107	95	95	92
avobenzone	82	91	***22***	100	97	86	***19***
azoxystrobin	98	107	94	104	98	94	95
BDE-99	93	91	97	102	94	98	85
benzophenone-1	83	94	82	105	96	94	88
BHC-β	106	121	110	127	103	117	104
BHT	116	118	120	117	108	119	109
bifenthrin	94	93	98	100	94	97	93
boscalid	96	102	96	104	98	94	82
caffeine	95	97	97	103	96	98	87
carbetamide	100	94	102	104	100	97	108
carbofuran	107	96	98	100	95	98	92
celestolide	110	107	109	111	103	116	104
chlordane-trans	100	100	104	108	95	105	97
chlorpyrifos	98	99	106	104	99	104	96
chlorpyrifos-methyl	101	105	111	109	103	109	97
climbazole	97	94	89	100	93	98	96
clopidogrel	97	97	99	102	96	100	94
clotrimazole	92	96	85	95	87	95	88
codeine	94	101	72	91	96	95	98
cybutryne	93	97	101	103	97	99	93
cyflufenamid	95	95	99	108	103	92	92
cyhalothrin-λ	94	94	100	97	91	97	93
DDE-*p,p'*	99	94	100	102	96	101	93
DEHA	95	84	97	99	94	96	94
DEHTP	94	77	97	100	98	95	91
deltamethrin	97	103	94	102	99	92	92
desmedipham	102	97	100	104	88	93	85
diazepam	97	96	94	101	95	95	92

Matrix: Liver	PSA	C18	Z-Sep®	chitin	chitosan	GCB	MWCNTs
dicofol-*o, p'*	97	100	106	105	99	103	***68***
dieldrin	101	98	91	99	97	99	90
difenoconazole	92	102	94	108	98	94	94
diflubenzuron	94	100	102	103	99	96	***24***
dimethomorph	98	100	98	108	104	96	92
diphenylamine	110	113	116	112	108	119	89
EHS	106	105	109	112	106	117	102
epoxiconazole	96	97	95	103	93	96	91
17 β-estradiol	96	108	95	99	94	96	86
17α-ethinylestradiol	95	99	99	102	96	96	95
fenhexamid	80	97	88	99	97	98	91
fenpropidin	96	99	87	102	94	97	95
fenpropimorph	95	99	96	103	96	101	98
fenvalerate	96	98	97	102	99	95	92
fipronil	95	101	101	106	95	100	100
fipronil sulfone	96	97	96	103	93	97	94
fluazifop-p-butyl	93	95	98	100	93	97	93
fludioxonil	95	96	96	100	94	98	84
fluopyram	93	97	101	103	95	97	94
fluorene	121	119	117	115	109	114	***24***
flupyradifurone	99	101	98	103	103	96	91
fluvalinat-τ	95	101	97	103	97	95	96
galaxolide	106	102	113	110	104	111	102
heptachlor	112	121	109	136	107	122	112
imidacloprid	125	101	94	101	88	126	105
isoproturon	89	103	99	103	89	95	86
lenacil	98	98	97	101	96	98	92
lilial	***68***	109	114	116	101	134	118
mestranol	94	96	97	102	93	95	95
metazachlor	95	103	99	106	93	97	93

Matrix: Liver	PSA	C18	Z-Sep®	chitin	chitosan	GCB	MWCNTs
metrafenone	95	96	96	98	93	100	92
miconazol	94	107	88	105	97	87	***50***
myclobutanil	99	96	95	101	95	97	92
octocrylene	96	96	96	102	95	97	96
4-*tert*-octylphenol	100	100	103	105	98	102	97
oxybenzone	94	98	86	104	98	101	76
PCB 101	93	92	100	103	94	106	97
PCB 138	97	93	97	97	95	97	93
PCB 153	94	87	95	103	95	97	93
PCB 180	94	85	98	102	91	94	92
PCB 28	106	104	118	112	110	120	90
PCB 52	101	100	106	108	103	110	98
pentachloronitrobenzene	124	115	108	123	108	118	105
permethrin	91	96	96	103	92	96	93
phenanthrene	113	110	115	113	109	87	***4***
picolinafen	95	96	97	102	93	94	***33***
pirimicarb	95	97	99	103	95	100	96
propiconazole	92	95	95	101	94	98	91
pyrene	95	91	100	101	96	48	***0***
pyrimethanil	96	98	99	103	97	90	***48***
quinoxyfen	94	91	96	101	93	94	***50***
simazine	107	101	97	102	95	94	87
spirodiclofen	96	96	104	107	99	96	92
TDCPP	97	97	96	102	95	96	94
tebuconazole	96	96	95	100	95	97	88
terbuthylazine	95	100	98	106	94	99	94
terbuthylazine-desethyl	90	105	102	104	97	95	90
terbutryn	94	92	97	101	95	100	94
testosterone	99	111	103	107	99	95	87
tetraconazole	94	97	98	103	95	99	92

Matrix: Liver	PSA	C18	Z-Sep®	chitin	chitosan	GCB	MWCNTs
thiabendazole	92	95	92	96	96	73	***7***
tolclofos-methyl	101	104	108	110	99	108	98
tonalid sum	104	102	110	109	100	109	102
triclosan	93	97	99	103	96	100	95
triphenyl phosphate	96	96	97	102	95	94	93
uvinul a plus	95	98	78	100	94	95	91
verapamil	101	106	89	101	96	93	94
zoxamide	98	120	95	113	96	94	84

Supplementary Material Table S3: List of all analytes, including classification, retention time (RT), applied MRM transitions (quantifier in **bold** letters) and collision energy (CE); API active pharmaceutical ingredients, PAH polycyclic aromatic hydrocarbons, POP persistent organic pollutants; [1]) Analytes with two or three isomers, all retention times given.

analyte	classification	RT [min]	precursor ion	product ion	CE [eV]
acenaphthene	PAH	5.98	153.0	126.0	35
			**153.0**	**77.0**	35
			153.0	51.0	45
atrazine	pesticide (herbicide)	7.64	215.0	200.1	5
			**215.0**	**58.1**	10
			200.0	94.1	15
avobenzone	UV blocker	15.87	310.1	295.2	10
			310.1	135.1	25
			**310.1**	**108.1**	10
azoxystrobin	pesticide (fungicide)	18.00	344.1	182.9	25
			**344.1**	**171.9**	40
			344.1	155.8	40
azoxystrobin-d_4	internal standard	17.99	407.0	348.0	5
			392.0	364.0	5
			**348.0**	**172.1**	35
BDE 99	POP	15.91	565.6	405.6	20
			**563.6**	**403.7**	20
			403.7	296.7	30
benzophenone-1	UV blocker	10.66	**213.0**	**128.1**	25
			213.0	77.0	40
			213.0	51.0	45
BHC-β	POP	7.74	218.9	183.1	5
			216.9	181.1	5
			**181.0**	**145.0**	15
BHT	antioxidant	5.92	220.2	205.2	10
			205.2	177.1	5
			**205.2**	**57.1**	10
bifenthrin		13.55	**181.2**	**165.2**	25

analyte	classification	RT [min]	precursor ion	product ion	CE [eV]
	pesticide (insecticide)		181.0	115.1	45
			166.0	115.1	35
boscalid	pesticide (fungicide)	16.20	**140.0**	**112.0**	10
			140.0	76.0	25
			111.9	76.0	15
caffeine	indicator for human pollution	8.51	194.0	109.1	10
			**194.0**	**55.0**	20
			109.0	55.0	5
carbetamide	pesticide (herbicide)	9.70	120.1	77.0	15
			119.1	91.0	15
			**119.1**	**64.1**	25
carbofuran	pesticide (insecticide)	7.56	164.0	149.1	5
			**164.0**	**103.1**	20
			149.0	121.1	5
celestolide	fragrance	7.38	**244.2**	**229.2**	5
			229.2	173.1	0
			229.2	57.1	10
chlordane-*trans*	POP	10.65	374.8	265.8	15
			**372.8**	**265.8**	15
			271.7	236.9	15
chlorpyrifos	pesticide (insecticide)	9.60	313.8	257.8	15
			**196.9**	**107.0**	40
			196.9	98.0	30
clorpyrifos-d_{10}	internal standard	9.54	325.9	262.1	10
			**323.9**	**260.0**	10
			259.8	167.0	15
chlorpyrifos-methyl	pesticide (insecticide)	8.80	287.9	92.9	20
			285.9	93.0	25

analyte	classification	RT [min]	precursor ion	product ion	CE [eV]
climbazole	API (antimycotic)	10.99	**124.9**	**47.0**	15
			**206.9**	**111.0**	20
			206.9	75.0	35
			180.0	125.0	5
clopidogrel	API (antiaggregant)	12.93	262.0	152.0	10
			**262.0**	**125.0**	25
			262.0	89.0	45
clotrimazole	API (antimycotic)	10.99	278.1	243.1	5
			**278.1**	**165.1**	25
			243.1	165.1	15
codeine	API (analgesic)	13.34	299.1	214.1	25
			**299.1**	**162.1**	5
			229.0	214.1	5
cybutryne	pesticide (fungicide)	10.36	253.0	196.1	15
			253.0	182.0	10
			**182.1**	**109.1**	5
cyflufenamid	pesticide (fungicide)	11.63	412.0	294.9	5
			188.1	88.0	35
			**118.1**	**90.0**	10
cyhalothrin-λ	pesticide (insecticide)	14.33	**208.0**	**181.0**	5
			208.0	152.0	25
			197.0	161.1	5
DDE-*p,p'*	POP (metabolite)	11.25	317.8	248.0	15
			315.8	246.0	15
			**246.1**	**176.2**	30
DEHA	plasticizer	12.85	147.0	55.1	15
			**129.1**	**101.1**	0
DEHTP	plasticizer	15.48	129.1	83.0	5
			261.1	149.0	5
			167.0	79.0	10
			**149.0**	**65.0**	10
deltamethrin	pesticide (insecticide)	17.73	**252.9**	**174.0**	0
			252.9	93.1	15
			251.0	172.0	0
desmedipham	pesticide (herbicide)	07.36	181.0	122.0	10
			**181.0**	**109.0**	10
			122.0	94.0	10
diazepam	API (anxiolytic)	11.34	256.1	221.2	5
			256.1	165.1	35
			**221.1**	**206.1**	35
dicofol-*o, p'*	POP	09.68	**139.0**	**111.0**	10
			139.0	75.0	30
			111.0	74.0	40
dieldrin	POP	11.34	277.0	241.0	5
			**262.9**	**193.0**	35
			262.9	191.0	35
difenoconazole[1]	pesticide (fungicide)	17.43	324.8	266.8	15
		17.50	**322.8**	**264.8**	15
			264.9	202.0	20
diflubenzuron	pesticide (insecticide)	5.00	**157.0**	**141.0**	5
			141.0	113.0	10
			141.0	63.0	25
dimethomorph[1]	pesticide (fungicide)	18.06	302.9	164.9	10
		18.33	**300.9**	**165.0**	10

analyte	classification	RT [min]	precursor ion	product ion	CE [eV]
diphenylamine	industrial chemical	06.70	300.9	138.8	15
			169.0	66.0	20
			**169.0**	**51.0**	45
			168.1	139.0	35
EHS	UV blocker	08.04	138.0	120.0	0
			**120.1**	**92.0**	5
			120.1	63.0	30
epoxiconazole	pesticide (fungicide)	13.23	**192.0**	**138.1**	10
			192.0	111.0	25
			138.0	75.0	25
17β-estradiol	API (hormone)	14.95	272.1	213.1	10
			**272.1**	**172.1**	5
			213.0	133.1	10
17α-ethinylestradiol	API (estrogen medication)	15.48	**296.1**	**213.1**	10
			213.1	133.1	10
			160.0	127.0	20
fenhexamid	pesticide (fungicide)	12.68	301.0	97.0	15
			179.0	115.0	15
			**177.1**	**113.0**	15
fenpropidin	pesticide (fungicide)	09.18	**273.0**	**98.0**	5
			145.0	117.0	10
			117.0	91.0	15
fenpropimorph	pesticide (fungicide)	09.57	128.1	110.1	5
			128.1	86.1	10
			**128.1**	**70.1**	10
fenvalerate	pesticide (insecticide)	17.21	419.1	166.8	10
			**167.0**	**125.1**	10

analyte	classification	RT [min]	precursor ion	product ion	CE [eV]
fipronil	pesticide (biocide)	10.35	167.0	89.0	40
			368.8	214.8	25
			**366.8**	**212.8**	25
			350.8	254.8	15
fipronil sulfone	pesticide (metabolite)	11.48	384.8	256.8	20
			**382.8**	**254.9**	20
			254.9	227.9	15
fluazifop-p-butyl	pesticide (herbicide)	11.64	382.9	282.0	10
			**281.9**	**238.0**	15
			254.0	146.1	15
fludioxonil	pesticide (fungicide)	11.24	248.0	182.1	10
			248.0	154.1	20
			**248.0**	**127.1**	30
fluopyram	pesticide (fungicide)	10.32	395.9	223.1	5
			**222.9**	**196.0**	10
			222.9	187.1	10
fluorene	PAH	06.53	166.0	115.0	35
			**165.0**	**115.1**	25
			165.0	63.0	45
flupyradifurone	pesticide (insecticide)	14.58	288.0	126.1	15
			128.0	90.0	10
			**126.0**	**73.0**	25
fluvalinate-*τ*[1]	pesticide (insecticide)	17.19	252.0	200.0	15
		17.25	**250.0**	**200.1**	15
			250.0	198.1	40
galaxolide	fragrance	08.49	**243.0**	**213.2**	5
			243.0	171.1	10

analyte	classification	RT [min]	precursor ion	product ion	CE [eV]
heptachlor	POP	08.98	243.0	155.1	35
			273.7	238.9	15
			273.7	236.9	15
			**271.7**	**236.9**	15
imidacloprid	pesticide (insecticide)	11.01	**211.0**	**113.0**	15
			126.0	89.9	5
			126.0	73.0	25
isoproturon	pesticide (herbicide)	04.46	161.1	146.1	5
			**146.2**	**128.1**	5
			146.2	77.0	20
lenacil	pesticide (herbicide)	12.66	233.9	153.1	5
			153.1	110.1	20
			**153.1**	**82.1**	20
lilial	fragrance	06.04	189.0	131.1	5
			189.0	115.1	35
			**189.0**	**91.1**	20
mestranol	API (estrogen medication)	15.09	**310.2**	**227.1**	10
			227.2	171.1	10
			227.2	147.1	10
metazachlor	pesticide (herbicide)	10.19	209.0	133.2	10
			**209.0**	**132.2**	15
			209.0	117.1	35
metrafenone	pesticide (fungicide)	14.96	**394.8**	**364.8**	15
			376.9	346.8	20
			226.9	169.0	10
miconazole	API (antimycotic)	17.64	**159.0**	**123.0**	15
			159.0	89.0	25

analyte	classification	RT [min]	precursor ion	product ion	CE [eV]
myclobutanil	pesticide (fungicide)	11.42	159.0	63.0	45
			**179.0**	**125.1**	10
			179.0	90.0	30
			150.0	123.0	15
octocrylene	UV blocker	15.03	249.1	204.1	5
			249.1	165.1	30
			**204.1**	**176.0**	25
4-*tert*-octylphenol	industrial chemical	06.54	**135.0**	**107.1**	10
			135.0	77.0	20
			135.0	51.0	40
oxybenzone	UV blocker	09.98	228.0	184.0	25
			**227.0**	**184.0**	20
			227.0	128.1	35
PCB 101	POP	10.75	327.9	255.9	30
			**325.9**	**255.9**	30
			253.9	184.0	35
PCB 138	POP	12.74	361.9	289.9	30
			**359.9**	**289.9**	30
			287.9	217.9	40
PCB 153	POP	12.24	361.9	289.9	25
			**359.9**	**289.9**	25
			287.9	217.9	40
PCB 180	POP	13.91	395.8	325.8	30
			393.8	358.8	15
			**393.8**	**323.8**	30
PCB 28	POP	08.70	258.0	186.0	25
			**256.0**	**186.0**	25

analyte	classification	RT [min]	precursor ion	product ion	CE [eV]
PCB 52	POP	09.26	186.0	151.0	25
			291.9	221.9	25
			**289.9**	**219.9**	25
			255.0	220.0	10
pentachloronitro-benzene	pesticide (fungicide)	07.91	294.8	236.8	15
			248.8	213.8	15
			**141.9**	**106.9**	30
permethrin[1]	pesticide (insecticide)	15.23	165.0	91.0	15
		15.36	**163.0**	**127.0**	5
			127.0	91.0	10
phenanthrene	PAH	8.02	**178.0**	**152.1**	20
			178.0	77.0	45
			89.0	76.0	5
picolinafen	pesticide (herbicide)	13.59	376.0	239.1	10
			**376.0**	**238.1**	20
			238.1	145.1	25
pirimicarb	pesticide (insecticide)	08.42	**238.0**	**166.2**	10
			166.0	71.1	25
			166.0	55.1	20
propiconazole[1]	pesticide (fungicide)	12.59	258.8	172.9	15
		12.71	172.9	109.0	30
			**172.9**	**74.0**	45
pyrene	PAH	10.80	202.0	174.1	45
			202.0	151.1	40
			**101.0**	**88.0**	5
pyrene-d_{10}	internal standard	10.80	**212.0**	**206.6**	30
			212.0	178.8	45

analyte	classification	RT [min]	precursor ion	product ion	CE [eV]
pyrimethanil	pesticide (fungicide)	8.00	106.0	91.8	5
			198.0	183.1	15
			198.0	158.1	20
			**198.0**	**118.1**	35
quinoxyfen	pesticide (fungicide)	12.55	306.8	237.0	20
			271.9	237.1	10
			**237.0**	**208.1**	30
simazine	pesticide (herbicide)	07.59	201.0	186.0	5
			**201.0**	**173.0**	0
			186.0	91.0	5
spirodiclofen	pesticide (acaricide)	15.27	**312.1**	**259.0**	10
			312.1	108.9	15
			157.0	73.0	25
TDCPP	flame retardant	12.48	381.0	158.9	5
			**209.0**	**99.0**	5
			191.0	74.9	5
tebuconazole	pesticide (fungicide)	12.93	250.0	125.0	20
			125.0	99.0	20
			**125.0**	**89.0**	15
terbuthylazine	pesticide (herbicide)	07.85	**229.0**	**173.0**	0
			214.0	104.0	20
			214.0	71.0	20
terbuthylazine-desethyl	pesticide (metabolite)	07.12	**186.2**	**104.0**	15
			145.1	110.1	10
			145.1	68.1	20
terbutryn	pesticide (herbicide)	09.29	**241.2**	**185.1**	0
			241.2	170.0	10

analyte	classification	RT [min]	precursor ion	product ion	CE [eV]
			185.1	170.0	0
			**288.2**	**124.1**	5
testosterone	API (hormone)	15.19	288.2	109.1	20
			246.1	185.1	5
			336.0	217.9	20
tetraconazole	pesticide (fungicide)	09.76	**336.0**	**203.8**	30
			170.9	136.0	10
			201.0	174.0	15
thiabendazole	pesticide (fungicide)	10.41	201.0	130.0	30
			**173.9**	**65.0**	30
			267.0	252.0	
tolclofos-methyl	pesticide (fungicide)	8.89	267.0	93.0	30
			267.0	63.0	45
			**326.0**	**325.0**	**5**
triclosan	API (disinfectant)	10.61	25.0	169.1	20
			235.0	77.0	35
		08.49	**258.0**	**243.2**	0
tonalid[1]	fragrance	08.57	243.0	187.1	0
			243.0	57.1	10
		13.00	**326.0**	**325.0**	5
triphenyl phosphate	flame retardant	08.57	325.0	169.1	20
			325.0	77.0	35
			**397.0**	**382.2**	10
uvinul a plus	UV blocker	18.17	382.0	280.1	10
			382.0	149.0	15
			303.1	260.2	5
verapamil	API (anti-arrhythmic)	19.13	303.1	151.1	15
			**303.1**	**58.0**	20
zoxamide	pesticide	13.19	259.9	189.0	10
	(fungicide)		**257.9**	**187.1**	10
			189.0	161.1	15
